# Supplementary material for: In the Absence of a TCR Signal IL-2/IL-12/18-Stimulated γδ T Cells Demonstrate Potent Anti-Tumoral Function Through Direct Killing and Senescence Induction in Cancer Cells
Source: Cancers (Basel). 2020 Jan 4;12(1):130. doi: 10.3390/cancers12010130 (PMC7017313; doi:10.3390/cancers12010130)
Supplement: Supplementary file 1 [file cancers-12-00130-s001.pdf]

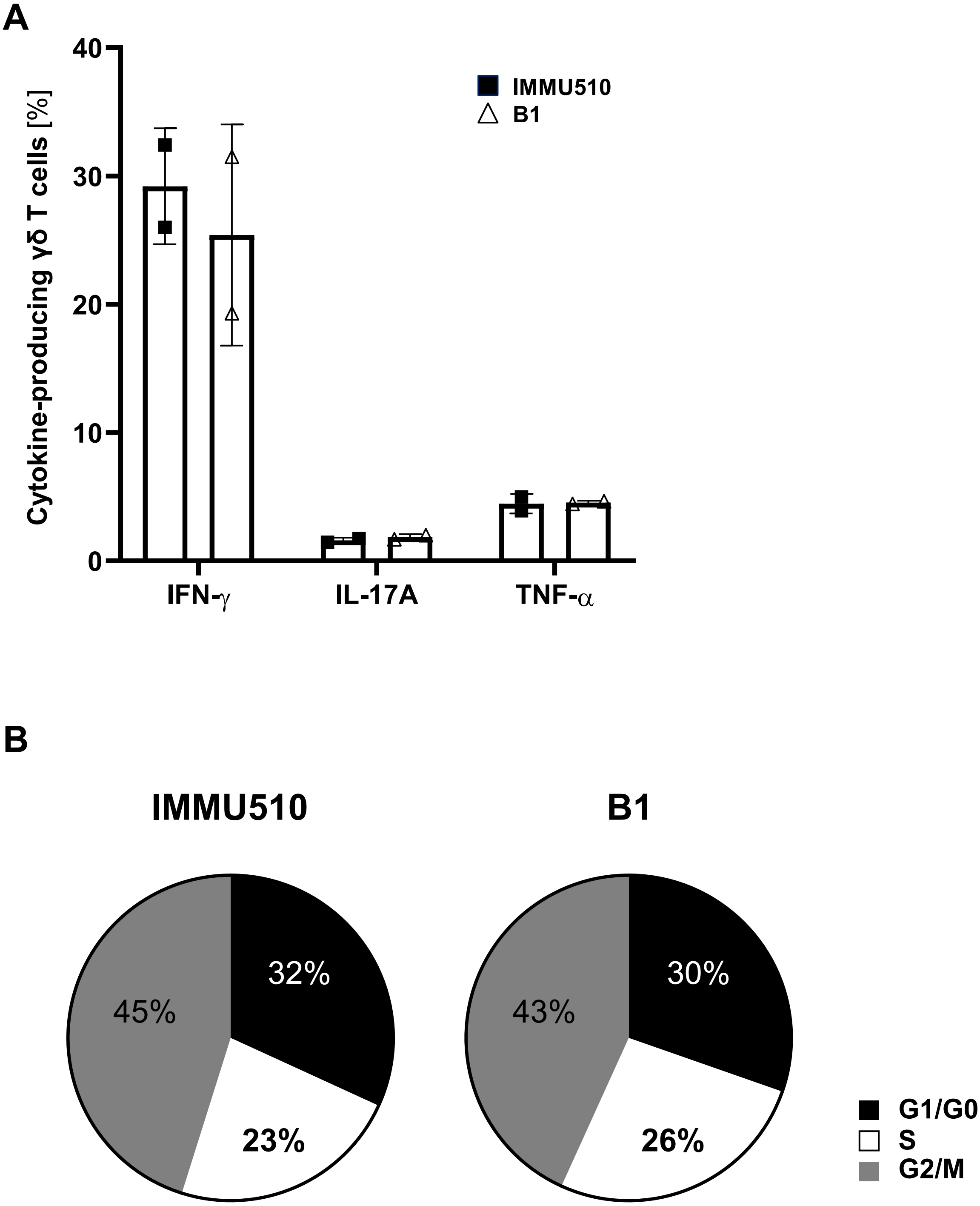

Supplementary Figure 1. Direct comparison of two different anti-γδTCR monoclonal antibodies, clone IMMU510 and clone B1. (A) isolated γδ T cells were stimulated with either IMMU510 or B1 together with IL-2/IL-12/IL-18. The frequencies of IFN-γ^{+}, IL-17A^{+}, and TNF-α^{+} γδ T cells were analyzed by flow cytometry. The data were obtained from 2 different healthy donors. (B) isolated γδ T cells were stimulated with either IMMU510 or B1 together with IL-2/IL-12/IL-18. These cells were co-cultured with Wm115 cells for 96 hours and cell cycle status of the tumor cells was evaluated by EdU-incorporation assay. Representative data from one donor are shown.
